# Computational and Biological Evaluation of *N*-octadecyl-*N*′-propylsulfamide, a Selective PPARα Agonist Structurally Related to *N*-acylethanolamines

**DOI:** 10.1371/journal.pone.0092195

**Published:** 2014-03-20

**Authors:** Inmaculada Moreno-Santos, Francisco Javier Pavón, Miguel Romero-Cuevas, Antonia Serrano, Carolina Cano, Margarita Suardíaz, Juan Decara, Juan Suarez, Fernando Rodríguez de Fonseca, Manuel Macías-González

**Affiliations:** 1 Unidad de Gestión Clínica de Endocrinología y Nutrición, Hospital Universitario Virgen de la Victoria, Instituto de Investigación Biomédica de Málaga (IBIMA), Málaga, Spain; 2 Centro de Investigación Biomédica en Red de Fisiopatología de la Obesidad y Nutrición (CIBERobn, CB06/03), Instituto de Salud Carlos III, Santiago de Compostela, Spain; 3 Unidad de Gestión Clínica de Salud Mental, Hospital Regional Universitario de Málaga, Instituto IBIMA, Málaga, Spain; 4 Grupo Moduladores de Receptores Cannabinoides y PPARs, Instituto de Química Médica, Centro de Química Orgánica “Manuel Lora-Tamayo” del Consejo Superior de Investigaciones Científicas (CSIC), Madrid, Spain; University of Bologna & Italian Institute of Technology, Italy

## Abstract

To further understand the pharmacological properties of N-oleoylethanolamine (OEA), a naturally occurring lipid that activates peroxisome proliferator-activated receptor alpha (PPARα), we designed sulfamoyl analogs based on its structure. Among the compounds tested, N-octadecyl-N′-propylsulfamide (CC7) was selected for functional comparison with OEA. The performed studies include the following computational and biological approaches: 1) molecular docking analyses; 2) molecular biology studies with PPARα; 3) pharmacological studies on feeding behavior and visceral analgesia. For the docking studies, we compared OEA and CC7 data with crystallization data obtained with the reference PPARα agonist GW409544. OEA and CC7 interacted with the ligand-binding domain of PPARα in a similar manner to GW409544. Both compounds produced similar transcriptional activation by *in vitro* assays, including the GST pull-down assay and reporter gene analysis. In addition, CC7 and OEA induced the mRNA expression of CPT1a in HpeG2 cells through PPARα and the induction was avoided with PPARα-specific siRNA. *In vivo* studies in rats showed that OEA and CC7 had anorectic and antiobesity activity and induced both lipopenia and decreases in hepatic fat content. However, different effects were observed when measuring visceral pain; OEA produced visceral analgesia whereas CC7 showed no effects. These results suggest that OEA activity on the PPARα receptor (e.g., lipid metabolism and feeding behavior) may be dissociated from other actions at alternative targets (e.g., pain) because other non cannabimimetic ligands that interact with PPARα, such as CC7, do not reproduce the full spectrum of the pharmacological activity of OEA. These results provide new opportunities for the development of specific PPARα-activating drugs focused on sulfamide derivatives with a long alkyl chain for the treatment of metabolic dysfunction.

## Introduction

The peroxisome proliferator-activated receptor α (PPARα) is a nuclear receptor involved in the control of lipid metabolism [1]. The large multifunctional ligand binding pocket of PPARα allows it to recognize a number of structurally heterogeneous molecules, both synthetic and natural. Synthetic PPARα agonists are low-affinity ligands of moderate selectivity, such as the fibrates, which are clinically used to treat blood lipid abnormalities [2], and high-affinity ligands, which are effective at reducing hyperlipidemia, atherosclerosis, and inflammation in animal models [3]–[4]. Among the several endogenous ligands proposed for PPARα, including non-esterified fatty acids, oxygenated fatty acids, and fatty acid ethanolamides or N-acylethanolamines (NAEs) [2], [5], N-oleoylethanolamine (also known as oleoylethanolamide or OEA) activates with high-potency PPARα-driven transactivation in a heterologous expression system with a half-maximal concentration (EC_50_) of 120 nM [5]–[6].

OEA is an oleoyl-derived (18∶1 cis-9) NAE that acts as a lipid mediator of satiety and exerts anorectic effects primarily through peripheral mechanisms with a discrete cerebral activation [7]. Although its effects on feeding appear to be mediated by PPARα [5], OEA has also been shown to be implicated in other activities, including cytoprotection, inflammation and pain, and may interact with other possible targets, such as vanilloid channels (TRPV1) or G protein-coupled receptors (e.g., GPR119) [8].

In the liver, the PPARα-mediated effects of OEA have been thoroughly investigated [9]. OEA has been reported to reduce the hepatic lipid content and its composition in diet-induced obese rats and wild-type mice but not in obese mice lacking the PPARα receptor gene [10]. These effects of OEA in the liver were accompanied by changes in the expression of PPARα and other PPARα-related genes, including stearoyl-CoA desaturase-1, which is a key enzyme involved in the synthesis of monounsaturated fatty acids and biosynthesis of hepatic cholesterol esters and triglycerides [11].

The molecular mechanism from OEA-dependent activation of PPARα to appetite inhibition is still poorly understood. PPARα may act by influencing the expression of satiety-inducing proteins, such as apolipoprotein A-IV [12]. However, the rapid onset of the OEA response (<30 min) and its reliance on intact vagal sensory innervations suggest the initial involvement of a transcription-independent signal that recruits sensory vagal afferents in the gut [7]. This signal remains unidentified, though the ability of PPARα to elicit rapid non-genomic responses has been documented [13]. In addition to metabolic activity, we have recently evaluated the effects of OEA on pain using PPARα-null and wild-type mice. Our data showed that OEA reduced visceral and inflammatory responses via a PPARα-independent mechanism [14]. However, there is little information on the physiological relevance of other OEA-activated G protein-coupled receptors, such as GPR119 [15], and the vanilloid receptor channel TRPV1 [16].

To clarify which actions of OEA are PPARα-dependent, we developed and characterized novel drugs that share biological activity with OEA, with some of them having greater potency for PPARα receptor than this endogenous ligand. We have reported recently that sulfamoyl and propyl-sulfamoyl derivatives with long chain saturated and unsaturated alkyl groups can act as PPARα activators [17]. Among the active compounds tested, N-octadecyl-N′-propylsulfamide (also referred to as CC7) has been identified as a potent appetite suppressant by acting as a concentration-dependent activator of PPARα [17].

However, the pharmacological profile of this compound has not been extensively explored in comparison with OEA. We have taken advantage of this sulfamide derivative to analyze the interaction of OEA and CC7 with the PPARα receptor using molecular docking and *in vitro* studies with co-activators. Complementary to these studies, we also performed *in vivo* characterizations of these PPARα ligands on feeding behavior, changes in body weight and lipid metabolism, as well as on visceral pain.

## Materials and Methods

### Animals

Food intake studies and metabolic evaluations were performed on 8–10 week-old male Wistar rats weighing 250–300 g. Additionally, adult male CD1 mice weighing 25–30 g were used for the analgesic studies. All animals were supplied by Charles River Laboratories España S.A. (Barcelona, Spain) and housed in the Animal Resource Center at the University of Málaga (Spain). Animals were kept in clear plastic cages under a 12 h light/dark cycle (lights off at 8∶00 PM) in a room at ambient temperature (23°C) and humidity (55%). Unless otherwise indicated, water and chow pellets were available *ad libitum* throughout the course of these studies.

### Ethics Statements

Experiments and procedures were conducted under strict adherence to the European Directive 2010/63/EU on the protection of animals used for scientific purposes and with Spanish regulations (RD 53/2013 and 178/2004). All efforts were made to minimize unnecessary suffering. All protocols were approved by the Committee on the Ethics of the *Hospital Regional Carlos Haya* (codes CVI-1038 and PI45403).

### Drugs

OEA [N-(cis-9-octadecenoyl)-ethanolamine] and GW7647 [2-(4-(2-(1-cyclohexanebutyl)-3-cyclohexylureido)ethyl)­phenyl­thio)-2-methyl­propionic acid] were purchased from Tocris Bioscience (Bristol, UK). The disubstituted sulfamides CC7 (N-octadecyl-N′-propylsulfamide) and CC12 (N-2-adamantyl-N′-propylsulfamide) were synthesized as previously described [17].

For *in vitro* molecular biology studies, the drugs were dissolved in dimethyl sulfoxide (DMSO). For *in vivo* studies in rodents, the drugs were dissolved in 20% Tocrisolve™-100 (Tocris Bioscience, Bristol, UK) and 80% physiological saline. OEA and CC7 were administered intraperitoneally (i.p.) at doses of 5 mg/kg in a volume of 1 mL/kg body weight in rats and 10 mL/kg body weight in mice. A solution of 0.6% acetic acid was prepared in physiological saline.

### Acute Treatment with OEA and CC7 for Feeding Behavior in Rats

The acute effects of OEA and CC7 on feeding behavior were analyzed in adult male Wistar rats (N = 7–8). After a short-term habituation, animals were deprived of food for 24 h but had free access to water [18]–[19]. Drugs were administered i.p. at a dose of 5 mg/kg at 15 min before the rats were given access to food. Animals were then returned to their individual home cages without any bedding material. Subsequently, a measured amount of food was placed into the cages (t = 0). Food pellets and food spillage were then weighed at time intervals of 30, 60, 120 and 240 min. The cumulative food intake (g/kg body weight) was calculated from these data.

### Subchronic Treatment with OEA and CC7 in Rats

#### Experimental design

To analyze the subchronic effects of OEA and CC7, free-feeding Wistar rats were divided into 3 experimental groups (N = 7) and treated for 7 days. These rats were injected daily with OEA (5 mg/kg), CC7 (5 mg/kg) or vehicle (20% Tocrisolve™-100 in saline). The amount of food eaten and the body weight were recorded daily.

#### Sample collection

At the end of the 7-day treatment with OEA and CC7, rats were anesthetized (50 mg/kg i.p. pentobarbital sodium) and euthanized by decapitation at 12 h after the final injection. Trunk blood samples were collected into tubes containing EDTA-Na_2_ (1 mg/mL blood). The blood was centrifuged (2,100×g for 10 min at 4°C), and the plasma was aliquoted. Immediately after the bleeding procedure, rats were dissected to collect the liver. Both samples were stored at −80°C until further biochemical analysis.

#### Lipid determination in the plasma

Plasma cholesterol and triglycerides were measured by the Hematology Service at the Hospital Regional Universitario de Málaga (Málaga, Spain) using a Hitachi 737 Automatic Analyzer (Hitachi Ltd., Tokyo, Japan).

#### Total fat determination in the liver

Total lipids were extracted from frozen liver samples according to the method reported by Bligh and Dyer [20] with chloroform-methanol (2∶1, v/v) and butylated hydroxytoluene (0.025%, w/v). After two centrifugation sessions (2,800×g for 10 min at 4°C), the lower phase containing the lipids was extracted using Pasteur pipettes. Nitrogen was used for drying each sample, and the liver fat content was expressed as a percentage of tissue weight.

### Acute Treatment with OEA and CC7 for Visceral Pain Model in Mice

The analgesic activity of OEA was measured using the writhing test as a rodent visceral pain model [14]. Nociception was induced by an i.p. injection of 10 mL/kg of 0.6% acetic acid solution in mice. The number of writhes was cumulatively counted over a 10 min period starting at 5 min after the administration of the acetic acid solution. A writhe was defined as a contraction of the abdominal muscles accompanied by an elongation of the body and extension of the hind limbs. Animals (N = 10–12 per group) were randomly selected to receive an i.p. injection of 5 mg/kg of OEA, 5 mg/kg of CC7 or the corresponding vehicle solution (20% Tocrisolve™-100 in saline) 15 min prior the acetic acid administration. Control animals received a similar volume of saline solution. Each animal was used once and received 1–2 doses of the drugs tested. All behaviors during the assay were recorded in a randomized manner by a blind observer.

### Computational Docking Studies

#### Theoretical calculations

All calculations were performed on an Intel® Core™2 Quad Workstation using Linux Ubuntu kernel 3.4.0.

#### Preparation of ligands and target macromolecules

Ghemical v2.10 software was used to build and optimize the structure of the ligands. Each molecule was optimized using the Tripos 5.2 force field and AM1 method consecutively until the energy gradient was less than 0.01 kcal/mol. ESP-fitted partial charges were calculated on optimized geometry at AM1 level. To prepare the appropriate file needed for the docking study, non-polar hydrogen atoms were merged, and rotatable bonds within the ligands were defined through the AutoDockTools v1.5 (ADT) program (The Scripps Research Institute: http://mgltools.scripps.edu/; accessed 22/06/2012) (La Jolla, CA, USA). The three-dimensional structure of PPARα ligand binding domain (LBD) was downloaded from the RCSB Protein Data Bank (Berman et al., 2000) (1K7L entry, chain A) (Xu et al., 2001). The ligands, salts, and water molecules were removed, and the tautomeric forms were checked. To optimize the hydrogen bond networks, the Mol Probity server was used to add hydrogen atoms (Davis et al., 2007). Finally, Kollman charges were computed through ADT.

#### Protocol for docking study

Docking experiments of the compounds were performed using the AutoDock v4 package [21]. For the calculations, a grid box (60×60×60 points) was constructed around the binding site based on the location of the co-crystallized ligand GW409544 (1K7L entry, chain A) (coordinates: X = −17.866; Y = −13.599; Z = −3.726). The axis dimensions were 22.5 Å, and the spacing of the grid points was 0.375 Å. [22] The Lamarckian genetic algorithm (LGA) procedure was employed, and the docking runs were set to 100, with the maximum number of generations set at 27,000 and the maximum number of energy evaluations set at 25,000,000. The remainder of the parameters was set to the default.

#### Analysis of the binding mode

To select the binding mode of each compound, we applied a qualitative analysis based on the location and orientation of the 100 most optimal docked conformations determined by AutoDock in relation to the co-crystallized ligand GW409544 [23]. Hydrogen bonds and other properties of ligand-receptor interaction of each compound were evaluated with the Accelrys Discovery Studio® v2.0 (Accelrys, Inc., San Diego, CA, USA). Measurements of docked conformations were carried out through ADT.

### Plasmids and DNA Constructs

#### Protein expression vectors

Full-length cDNAs for the wild-type human PPARα, human retinoid X receptor alpha (RXRα) and human SRC1 were subcloned into the T7/SV40 promoter-driven pSG5 expression vector (Stratagene-Agilent Technologies Inc., Santa Clara, CA, USA), whereas the full-length cDNA of the mouse nuclear receptor co-repressor (NCoR) was subcloned into the T7/CMV promoter-driven pCMX expression vector. The same constructs were used for both T7 RNA polymerase-driven *in vitro* transcription/translation of the respective cDNAs and viral promoter-driven overexpression of the respective proteins in mammalian cells.

#### GST fusion protein constructs

The nuclear receptor interaction region of the human steroid receptor co-activator 1 (SRC1) spanning from amino acid 597 to 791 was subcloned into the pGEX glutathione S-transferase (GST) fusion vector (Amersham Biosciences-GE Healthcare Europe GmbH, Barcelona, Spain) [24]. This fragment was then subcloned into the *Bam*HI/*Eco*RI sites of the pGEX-2T plasmid.

#### Reporter gene constructs

Four copies of the human CPT1 gene DR1-type PPRE [CPT1 DR1-PPRE] (core sequence 5′-GTAGGGAAAAGGTCA-3′) were individually fused with the thymidine kinase (*tk*) minimal promoter driving the firefly luciferase reporter gene.

### In vitro Translation and Bacterial Overexpression of Proteins

Translated wild type PPARα and RXRα, as well as SRC1 (spanning amino acids 597–791) and NCoR (spanning amino acids 2218–2453 and including the second receptor interaction domain) proteins [25]–[27], were generated by coupled *in vitro* transcription/translation using rabbit reticulocyte lysate as recommended by the manufacturer (Promega Co., Madison, WI, USA). Protein batches were quantified by test translations in the presence of [^35^S]-methionine. The specific concentration of the receptor proteins was adjusted to 4 ng/μL after taking the individual number of methionine residues per protein into account. Bacterial overexpression of either GST-SRC1 or GST alone was obtained from the *E. coli* BL21 (DE3) pLysS strain (Stratagene-Agilent Technologies Inc., Santa Clara, CA, USA) containing the respective expression plasmids [26], [28]. Overexpression was stimulated with 0.25 mM isopropyl-β-D-thiogalactopyranoside for 3 h at 37°C followed by purification and immobilization on glutathione-Sepharose 4B beads (Amersham Biosciences-GE Healthcare Europe GmbH, Barcelona, Spain) according to the manufacturer’s protocol. Proteins were eluted in the presence of glutathione.

### Isolation of Protein Extracts

To analyze the protein levels in livers from subchronically-treated rats with OEA and CC7, approximately 50 mg of frozen livers were lysed with NE-PER nuclear and cytoplasmic extraction reagents from Pierce Biotechnology Inc. (Thermo Fisher Scientific, Rockford, IL, USA) and homogenized using a Silent Crusher S homogenizer (Heidolph Instruments GmbH & Co, Schwabach, Germany). After a 5 min incubation at 4°C, lysates were clarified by centrifugation (16,000×g for 10 min at 4°C) into a cytoplasmic supernatant and a nuclear pellet, the latter of which was treated to form the nuclear extract, and quantified (Bio-Rad Laboratories Inc., Hercules, CA, USA).

### GST Pull-down Assays

GST pull-down assays were performed with 50 μL of a 50% Sepharose bead slurry of GST-vector and GST-SRC1 (blocked with 1 μg/μL BSA) and 20 ng *in vitro*-translated, [^35^S]-labeled PPARα in either the presence or absence of different compounds [28]. Proteins were incubated in immunoprecipitation buffer [20 mM HEPES (pH 7.9), 200 mM KCl, 1 mM EDTA, 4 mM MgCl_2_, 1 mM DTT 0.1% Nonidet P-40 and 10% glycerol] for 20 min at 30°C. Proteins translated *in vitro* that were not bound to the GST-fusion proteins were washed away with immunoprecipitation buffer. [^35^S]-labeled PPARα that was bound to the GST-fusion protein was resolved by electrophoresis in a 15% sodium dodecyl sulfate-polyacrylamide gel and quantified on a Fuji FLA-3000 reader using Image Gauge software (Fujifilm Co., Tokyo, Japan).

### Gel Shift and Super-shift Assays

Gel shift assays were performed with equal amounts (10 ng) of appropriate *in vitro*-translated proteins and nuclear extracts (5–10 μg). The proteins were pre-incubated on ice for 15 min in a total volume of 20 μL binding buffer [10 mM HEPES (pH 7.9), 150 mM KCl, 1 mM DTT, 0.2 μg/μL poly (dI-C) and 5% glycerol].

For super-shift assays, either 1 μL of an antibody directed against the PPARα antibody (Santa Cruz Biotechnology, Inc., Santa Cruz, CA, USA) or 5–10 μg of bacterially expressed GST-SRC1 (or GST alone as a negative control) were added to the reaction mixture [27]. Then, 1 ng of the [^32^P]-labeled double-stranded oligonucleotides (50,000 cpm) corresponding to one copy of the human CPT1 (DR1-type RE) and acyl-CoA oxidase ACOX (PPRE) genes were then added, and incubation was continued for 15 min at room temperature [29]. Protein-DNA complexes were resolved by electrophoresis on 8% non-denaturing polyacrylamide gels in 0.5×TBE [45 mM Tris, 45 mM boric acid, 1 mM EDTA (pH 8.3)]. The gels were dried and quantified on a Fuji FLA3000 reader using Image Gauge software (Fujifilm Co., Tokyo, Japan).

### Transfection and Luciferase Reporter Gene Assays

MCF-7 human breast cancer cells were seeded into 6-well plates (200,000 cells/well) and grown overnight in phenol red-free Dulbecco’s modified Eagle’s medium (DMEM) supplemented with 5% charcoal-stripped fetal bovine serum. Plasmid DNA containing liposomes were formed by incubating 1 μg of expression vectors for wild type human PPARα, RXRα and SRC1 (pSG5) or NCoR (pCMX) and 1 μg of reporter plasmid CPT1 with 10 μg of N-[1-(2,3-Dioleoyloxy)]-N,N,N-trimethylammonium propane (DOTAP) from Roche Applied Science (Basel, Switzerland) for 15 min at room temperature in a total volume of 100 μL. After dilution with 900 μL phenol red-free DMEM, the liposomes were added to the cells. Phenol red-free DMEM supplemented with 15% charcoal-stripped fetal bovine serum was added 4 h after transfection. At this time, the compounds were also added. The cells were lysed 16 h after the onset of stimulation using the reporter gene lysis buffer (Roche Applied Science, Basel, Switzerland), and the constant light signal luciferase reporter gene assay was performed as recommended by the manufacturer. The luciferase activities were normalized with respect to the protein concentration.

### Gene Silencing with Small Interfering RNA (siRNA)

HepG2 (liver hepatocellular carcinoma cell line) (ATCC, HB-8065) cells were cultured following the manufacturer’s guidelines. Cells were treated for 48 h with the vehicle (DMSO) or growing concentrations of OEA (0.1–10 μM). Cells were serum deprived for 16 h before stimulation with the vehicle (DMSO) or 10 μM of OEA or CC7 for 48 h.

The siRNA targeting human PPARα was purchased from Dharmacon Inc. (Thermo Fisher Scientific, Lafayette, CO, USA) and two sequences (D-003434-01, D-003434-02) were used simultaneously according to the manufacturer’s instructions. The siRNA negative control from Dharmacon (D-001810-01) was used to test non-specific effects on gene expression. Overnight starved HepG2 cells were transfected using the transfection reagent Nucleofector kit and the Nucleofector II Device (Amaxa-Lonza Group Ltd., Basel, Switzerland) according to the manufacturer’s instructions in 6-well plates containing 100,000 cells/well with 5 nM siRNA per well (each sequence 2.5 nM). 30 min after start of transfection, cells were treated for 48 h with OEA and CC7 (10 μM; 0.5% serum) before mRNA analysis.

### RNA Isolation and Quantitative Real-time PCR Analyses

RNA from the HepG2 cells was extracted using the Trizol® method, according to the manufacturer’s instruction (Gibco BRL Life Technologies, Baltimore, MD, USA). To ensure the purity of the mRNA sequences and exclude proteins and molecules smaller than 200 nucleotides, RNA samples were isolated with an RNeasy Minelute Cleanup Kit (Qiagen, Hilden, Germany), which included digestion with DNase I column (RNase-free DNase Set, Qiagen), according to the manufacturers’ instructions. The total mRNA concentrations were quantified using a spectrophotometer (Nanodrop 1000 Spectrophotometer, Thermo Scientific, Rochester, NY) to ensure A260/280 ratios of 1.8 to 2.0.

Reverse transcription was performed from 1 mg of mRNA using the Transcriptor Reverse Transcriptase kit and random hexamer primers (Transcriptor RT, Roche Diagnostic GmbH, Mannheim, Germany). Negative controls included reverse transcription reactions that omitted the reverse transcriptase. Quantitative real-time reverse transcription polymerase chain reaction (qRT-PCR) was performed using an ABI PRISM® 7300 Real-Time PCR System (Applied Biosystems, Foster City, CA, USA) and the FAM dye label format for the TaqMan® Gene Expression Assays (Applied Biosystems) [30]. Absolute values from each sample treated with different compounds or OEA concentrations were normalized with regard to the housekeeping gene *Gapdh*. The relative quantification was calculated using the ΔΔCt method and normalized to the control or vehicle treatment. Primer of *Cpt1a* was obtained based on Primer references for TaqMan® Gene Expression Assays Applied Biosystem (Assay ID: Rn00580702_m1; Amplicon length: 64).

### Statistical Analyses

All of the data for the graphs and tables are expressed as the mean ± SEM. The various *in vivo* experiments included 7–8 animals in each group based on the assay used. The significance of differences between groups was evaluated with respect to control groups by two-way analysis of variance (ANOVA) followed by *post hoc* analysis for multiple comparisons (Bonferroni’s test). Student’s t-tests were performed for the *in vitro* studies (Welch’s correction was applied when variances were not equal). The statistical analysis of results was performed using the computer program GraphPad Prism v5.04 (GraphPad Software Inc., San Diego, CA, USA). A P-value less than 0.05 was considered to be statistically significant. See Supporting Information (Material and Methods S1).

## Results

### OEA Induces Ligand-dependent PPARα Complex Formation with DNA

**Figure 1:**
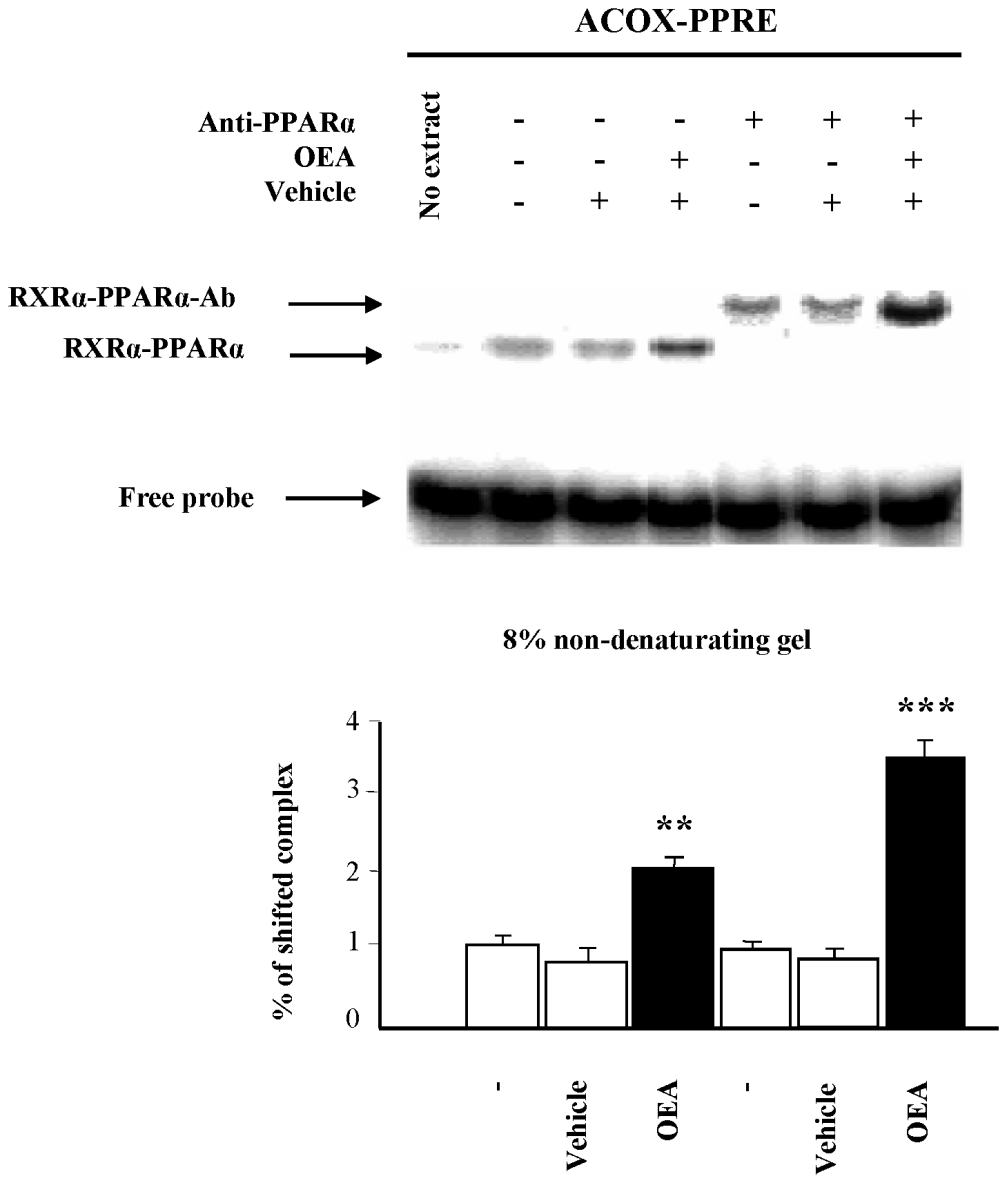
OEA induces ligand-dependent PPARα complex formation on DNA. The electrophoretic mobility shift assay (EMSA) was performed with hepatic nuclear extracts of rats treated with OEA and/or vehicle and [^32^P]-labeled rat acyl-CoA oxidase (ACOX)-response element (PPRE). For super-shift experiments, 1 μL of anti-PPARα antibody was added. Protein-DNA complexes (RXRα-PPARα and RXRα-PPARα-Ab) were separated from free probe on 8% non-denaturing polyacrylamide gels. Representative gels are shown. The first lane of each gel represents the probe alone as a negative control; all of the other lanes contained nuclear extract. The percentage of protein complexed with DNA was quantified after 6 determinations. The data are represented as the mean ± SEM and were analyzed by Student’s t test. **P<0.01 and ***P<0.001 compared to the vehicle group.

As previously demonstrated with other PPARα agonists (including CC7), we wanted to show the ability of OEA to induce the conformational changes that promote PPARα-RXRα heterodimerization and subsequent binding to DNA by mobility gel shift assay from hepatic nuclear extracts. We showed that PPARα-RXRα heterodimers bind to ACOX-PPRE, even in the absence of ligand (**Fig. 1**). As expected, OEA enhanced the ability of the interaction between the PPARα-RXRα heterodimer and PPRE by 2-fold compared to nuclear extracts from the non-treated control group (P<0.01). Because the large number of transcription factors that can bind to DR-1 elements could complicate the interpretation of this experiment, super-shift assays were also performed. The amount of super-shifted complex significantly increased by more than 3-fold upon co-administration of OEA (P<0.001). The super-shift assays showed that OEA specifically activated PPARα in the liver, but vehicle alone did not affect this activation.

### Docking of the Compounds in PPARα Receptor

**Figure 2:**
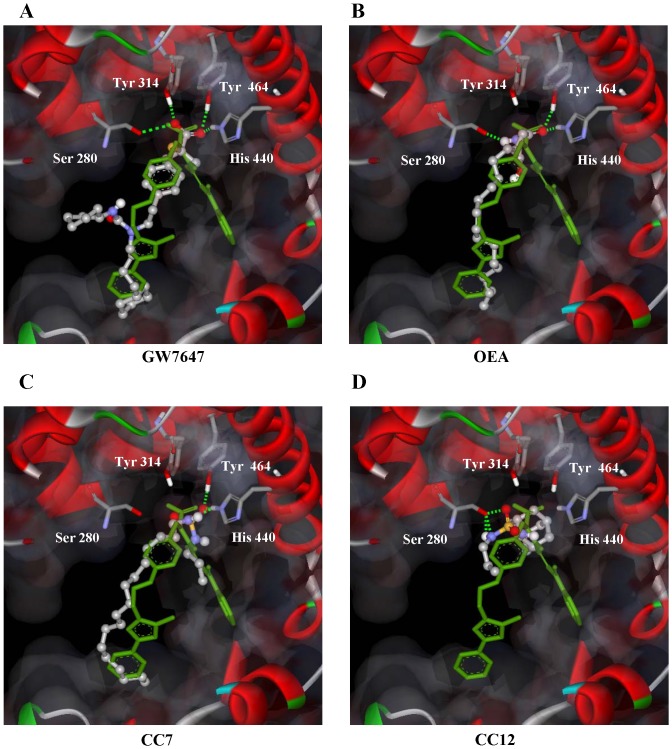
Computational docking representation of the binding modes of the optimal conformation/orientation in PPARα-LBD for GW7647, OEA, CC7 and CC12. (**A**) GW7647 pose #62, (**B**) OEA pose #45, (**C**) CC7 pose #36 and (**D**) CC12 pose #58 in ball and stick format colored by atom type. The co-crystallized conformation of GW405544 is shown in green, and the protein backbone is represented by ribbons (blue). The critical polar interactions (green line) and residues involved (Ser-280, Tyr-314, His-440 and Tyr-464) are shown. All agonist compounds formed a hydrogen bond with Tyr-464. The most ideal docking pose (#58) of CC12 only interacts with hydroxyl group of Ser-280.

Docking experiments of the 4 experimental compounds (OEA, CC7, CC12 and GW7647) were performed by means of the AutoDock v4 package. Validation of the *in silico* methods was achieved by comparing theoretical binding models with the RX experimental conformation of GW409544 (PDB: 1K7l) bound to the ligand binding domain of PPARα (PPARα-LBD) [30]. The 100 most optimal docked conformations of each compound were selected and analyzed. We observed that conformation #62 of GW7647 (grey), another commercial PPARα ligand, aligns perfectly with the crystallographic orientation of GW409544 (green) by forming the four polar interactions described previously to keep the helix 12 closed (Tyr-464, His-440, Tyr-314 and Ser-280) (**Fig. 2A**). We also observed that the docking representation of the best binding mode of OEA, #45 (grey), aligned well with the profile of the GW409544 (green) by forming three of the four described polar interactions (no interaction with amino acid Tyr-314) (**Fig. 2B**). Similarly, we observed that conformation #36 of CC7 (grey), the sulfamoyl analog of OEA, (**Fig. 2C**) formed two hydrogen bonds with the amino acids His-440 and Tyr-464. Thus, all of the conformations with the ligands have a structural profile similar to that observed with GW409544. The most important interaction of all of these compounds occurs with Tyr-464 by facilitating the interaction with co-activators. This is supported by notion that the docking model of the inactive compound CC12 did not show any evidence of this molecule interacting with Tyr-464 (**Fig. 2D**).

Finally, **Table 1** summarizes the number of likely conformations, the percentage of hydrogen bonds with the hydroxyl group of Tyr-464 of the 100 most optimal docked conformations and the docking energy for the selected orientation of each compound analyzed. These results confirmed that GW7647, OEA and CC7 could bind to PPARα better and with more stability than CC12. We then investigated if OEA and CC7 could act as agonists at PPARα by structural modeling.

**Table 1:** Docking of compounds in the ligand binding domain of PPARα.

Compound	Docking Energy (kcal mol^−1^)	Tyr 464 HB
**GW409544**	−18.25	31
**GW7647**	−16.03	27
**OEA**	−13.54	28
**CC7**	−14.01	26
**CC12**	−10.56	0

Docking energy (kcal/mol) and the percentage of the occurrence of hydrogen bonds (HB) to the hydroxyl group of Tyr 464 residue.

### Individual Ligand-enhanced Interaction of PPARα with Co-activators in Solution and Ligand-dependent Interaction of PPARα-RXRα with Co-activators on DNA

**Figure 3:**
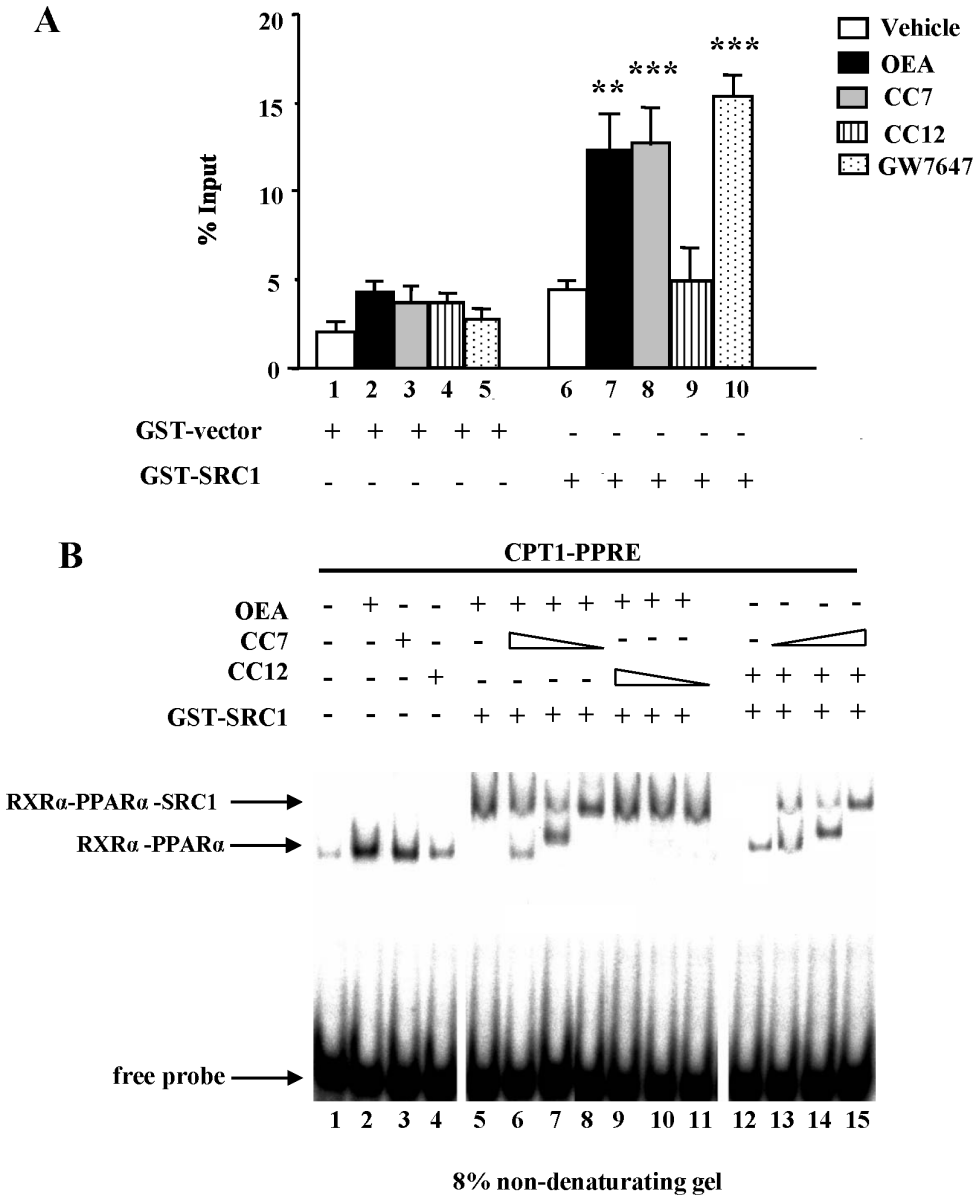
Ligand-dependent interaction profiles between the PPARα and co-activators and between PPARα-RXRα heterodimers and co-activators on DNA. (**A**) GST pull-down assays performed with GST-SRC1_597–791_ and [^35^S]-labeled wild-type PPARα in the presence of vehicle (DMSO), OEA (1 μM), CC7 (1 μM), CC12 (1 μM) and GW7647 (1 μM). The percentage of precipitated PPARα proteins with respect to input was quantified. The columns represent the mean ± SEM of at least three experiments, and the data were analyzed by Student’s t test. **P<0.01 and ***P<0.001 compared to SRC1 interaction in presence of vehicle alone. (**B**) Ligand-dependent interaction profiles of PPARα-RXRα heterodimers with co-activators on DNA. Combined gel shift and super-shift experiments were performed with wild type PPARα-RXRα protein and [^32^P]-labeled human CPT1-PPRE. PPARα-RXRα heterodimers were pre-incubated with solvent and different concentrations (1, 5 and 10 μM) of OEA, CC7 and CC12 followed by the addition of 2 μg of either GST alone (as a control) or GST-SRC1_597–791_. Protein-DNA complexes were resolved from the free probe with 8% non-denaturing polyacrylamide gels. Representative experiments are shown.

The ligand-dependent physical interaction between the PPARα and the co-activator SRC1 was assessed by transferase GST pull-down assays (**Fig. 3A**). For this purpose, *in vitro*-translated, [^35^S]-labeled wild-type PPARα protein was incubated in the presence of various compounds (OEA, CC7, CC12 and GW7647) and either GST alone or GST-SRC1 fusion protein immobilized on Sepharose beads. Samples incubated with GST protein alone showed weak residual association with PPARα (**bars 1–5**), which was considered to be nonspecific binding. The affinity of apo-PPARα in solution for co-activators could be explained by the significant molar excess of bacterially produced co-activator fusion proteins. However, classical endocrine nuclear receptors show low basal activity in the absence of ligand, whereas adopted orphan nuclear receptors such as PPARα display a significant amount of constitutive activity [26]. Here, PPARα showed reasonable levels of association with SRC1 in the absence of ligand (**bar 6**), which were increased significantly by OEA (P<0.01, **bar 7**), CC7 (P<0.001, **bar 8**) and GW7647 (P<0.001, **bar 10**) treatments. No interaction was observed between PPARα and SRC1 in presence of CC12 in solution (**bar 9**).

To support the results showing that the sulfamoyl analog of OEA enhanced the interaction between PPARα and SRC1, we measured the amount of DNA-bound heterodimer complexes with RXRα. Super-shift assays (**Fig. 3B**) were performed using *in vitro*-translated PPARα-RXRα heterodimers, bacterially produced NR interaction domains of the p160-CoA family members SRC1 and a [^32^P]-labeled double-stranded oligonucleotides with the sequence of the human CPT1-PPRE. The addition of either OEA or CC7 induced an increase in PPARα-RXRα complex formation on DNA (**lanes 2 and 3**). However, solvent alone (**lane 1**) and CC12 (**lane 4**) were unable to induce the formation of the PPARα complex on DNA. Interestingly, DNA-bound PPARα was able to attract a significant amount of the co-activator protein SRC1 after the conformational change induced by OEA (**lane 5**). The compound CC12 did not induce any interaction of PPARα with co-activator protein (**lane 12**), and a combination of OEA and CC12 resulted in a strong association with the co-activator (**lanes 9–11**). However, the combination of CC7 and CC12 resulted in the interaction of a significant level of SRC1 with DNA-bound PPARα-RXRα at high concentrations of both ligands (**lanes 13–15**) similar to what was observed with the combination of CC7 and OEA (**lanes 6–8**). Solvent alone did not induce any super-shift with GST-SRC1 (data not shown). These results suggest that PPARα is able to interact with SRC1 in the presence of OEA and CC7.

### Agonistic Action of PPARα Ligands

**Figure 4:**
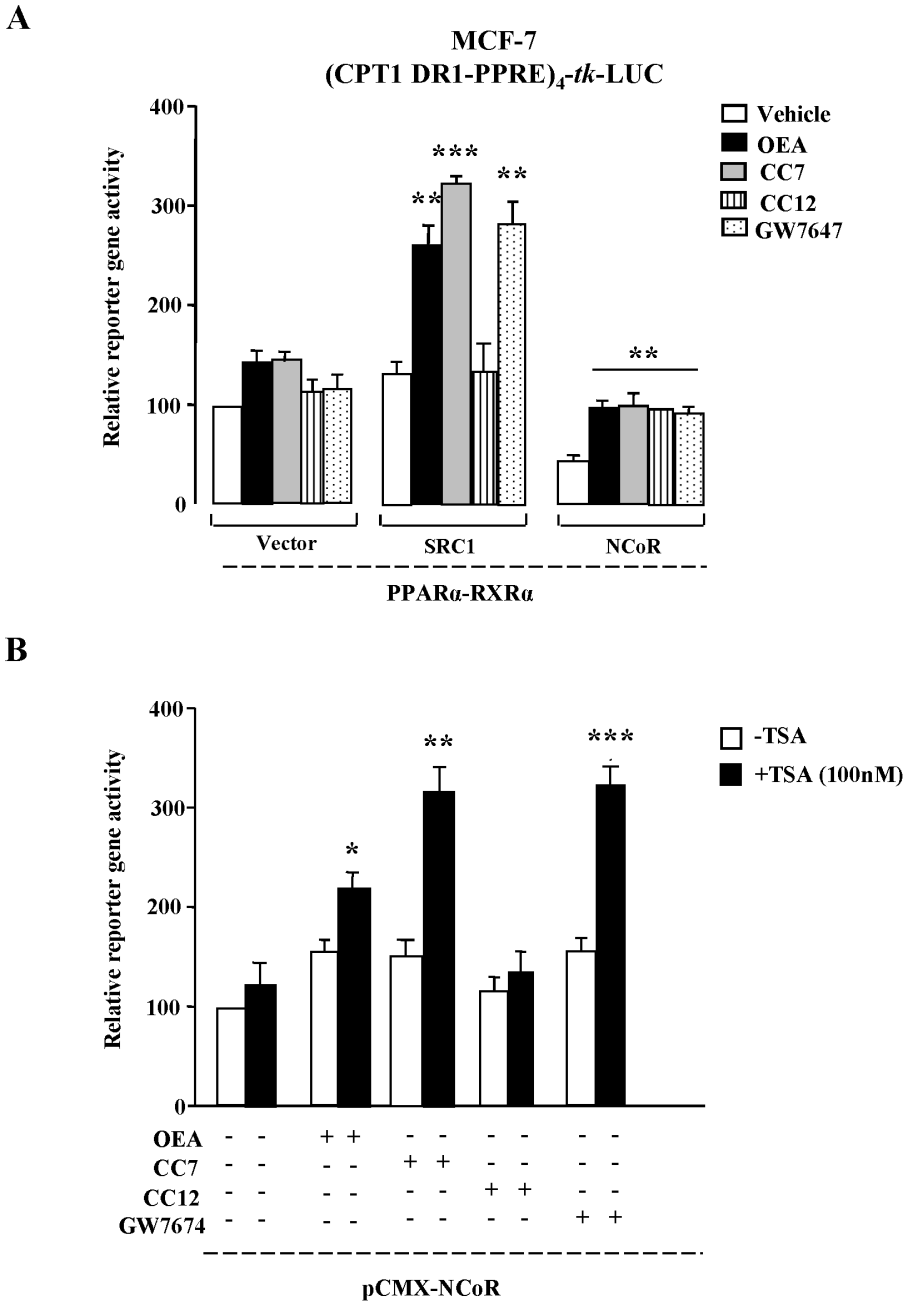
Basal and ligand-induced activities of PPARα were determined using luciferase reporter gene assays. (**A**) MCF-7 cells were transiently transfected with a reporter construct containing the human CPT1 DR1-type PPRE (CPT1-PPRE) and the indicated expression vectors for PPARα, RXRα, SRC1 (co-activator) and NCoR (co-repressor). The columns represent the mean ± SEM of at least three experiments, and the data were analyzed by Student’s t test. **P<0.01 and ***P<0.001 compared to vehicle alone. (**B**) MCF-7 cells were treated for 16 h with 10 μM of OEA, CC7, CC12 or GW7647 in either presence or absence and of 100 nM of the HDAC inhibitor trichlorostatin A (TSA). Stimulation of luciferase activity was normalized to the basal activity of PPARα-RXRα in absence of ligands. The columns represent the mean ± SEM of at least three experiments, and these data were analyzed by Student’s t test. *P<0.05, **P<0.01 and ***P<0.001 compared to the absence of TSA and the presence of vehicle.

To further explore the agonistic action of CC7, we performed reporter gene assays from extracts of MCF-7 cells that were transiently transfected with a luciferase reporter gene construct under the control of four copies of the human CPT1-RE fused with the *tk* promoter (**Fig. 4**). The first assay studied the influence of the overexpression of PPARα, RXRα and SRC1 on CPT1-RE-driven reporter gene activity (**Fig. 4A**). Stimulation with OEA, CC7 and GW7647 resulted in no changes in the basal activity compared to vehicle treatment. In fact, overexpression of SRC1 caused further induction of the basal activity and a strongly increased OEA, CC7 and GW7647 activation by 2-fold, which were significant compared to vehicle treatment (P<0.01; P<0.001; P<0.01; respectively). However, the presence of the nuclear receptor co-repressor (NCoR) reduced the overall basal reporter gene activity, but all of the ligands induced a significant increase (P<0.01) compared to vehicle treatment.

To investigate if PPARα modulates the acetylation status of histones or other factors associated with CPT1-PPRE target genes, MCF-7 cells transfected with the PPARα and RXRα were treated with OEA, CC7, CC12 and GW7647 in either the presence or absence of the HDAC inhibitor TSA (**Fig. 4B**), which can bypass the function of NCoR. PPARα inhibition was significantly abolished in cells treated with OEA, CC7 and GW7647 (P<0.05, P<0.01 and P<0.001, respectively) when TSA was added to block HDAC activity. However, TSA treatment had no effect on the NCoR repression of PPARα signaling in presence of CC12. Taken together, these assays suggest that CC7 acts in a similar manner to OEA and rule out the notion of enhanced recruitment of HDACs as the mechanism of PPARα inhibition in presence of NCoR.

### OEA and CC7 Induced the mRNA Expression of a PPARα Target Gene in Human HepG2 Hepatocytes

**Figure 5:**
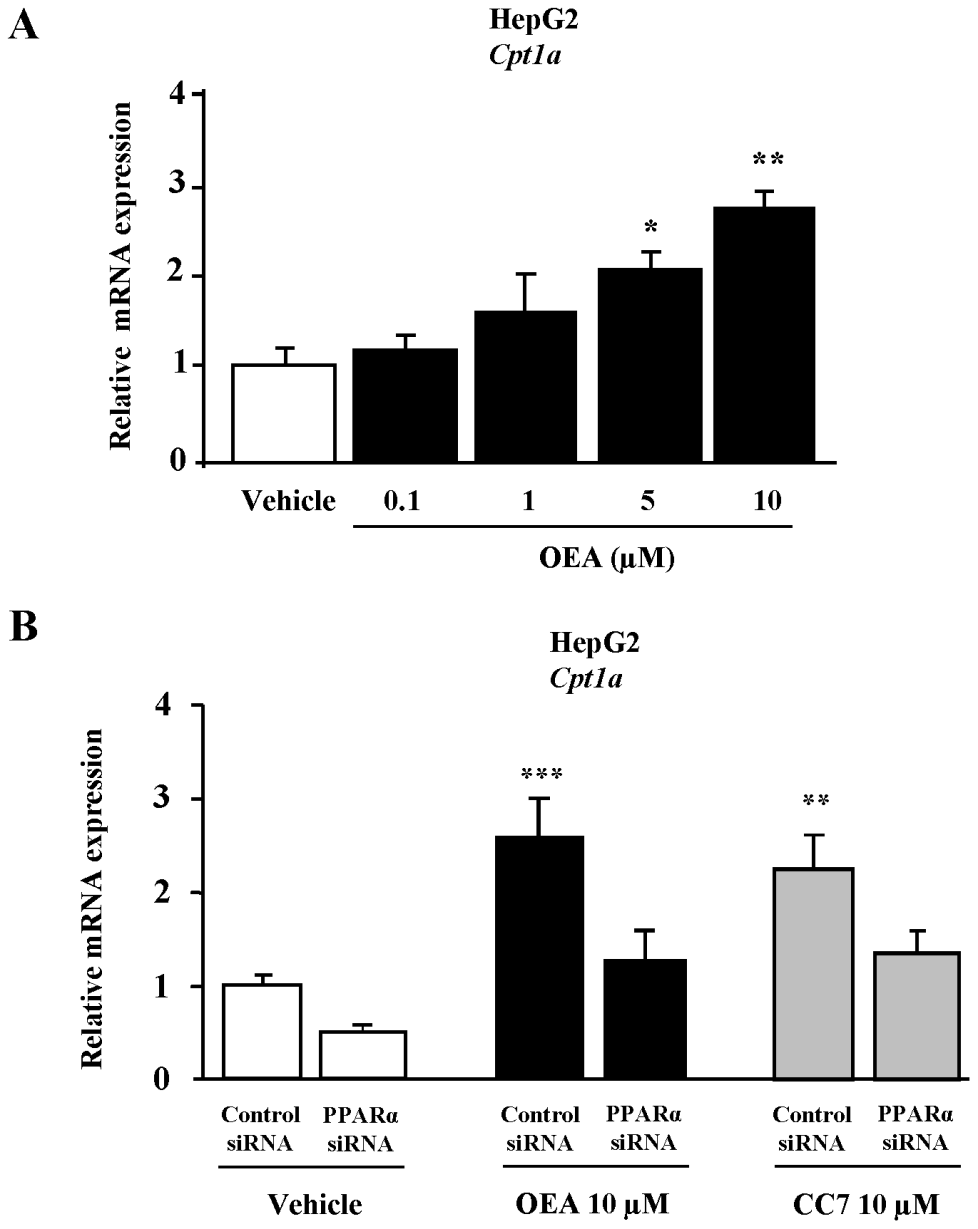
mRNA expression of CPT1a in HepG2 cells. (**A**) The CPT1a mRNA expression was determined by qRT-PCR after the incubation of cells with OEA (0.1, 1, 5 and 10 μM) for 48 h. (**B**) HepG2 cells were serum deprived for 16 h and transfected with PPARα-specific siRNA or control siRNA followed by incubation with 10 μM of OEA and CC7 for 48 h. Then, the CPT1a mRNA was determined by qRT-PCR. Expression was normalized to GAPDH expression. Experiments were repeated 3 times and results are presented as x-fold induction over vehicle treated cells. Columns represent the mean ± SEM, and these data were analyzed by Student’s t test. *P<0.05, **P<0.01 and ***P<0.01 vs vehicle or control siRNA.

To evaluate whether OEA regulates classical PPARα target genes, mRNA expression of CPT1a, the rate limiting enzyme of fatty acid oxidation, was examined in HepG2 cells. OEA induced CPT1a mRNA expression after 48 h in a dose-dependent manner. In fact, at 10 μM of OEA, mRNA expression was significantly increased (2.9-fold relative to vehicle treated cells, P<0.01) (**Fig. 5A**). To demonstrate that OEA mediates these inductive actions of CPT1a mRNA via PPARα activation, HepG2 cells were transfected with PPARα-specific siRNA or control siRNA. After PPARα siRNA transfection in HepG2 cells, the CPT1a mRNA expression was unaltered in the presence of OEA. Similarly, 10 μM of CC7 also induced a significant increase of CPT1a mRNA (P<0.01) that was abolished after the transfection of PPARα-specific siRNA(**Fig. 5B**). Therefore, the induction of CPT1a mRNA expression in the presence of OEA and CC7 was mediated by PPARα.

### Acute Effects of OEA and CC7 on Feeding Behavior in Wistar Rats

**Figure 6:**
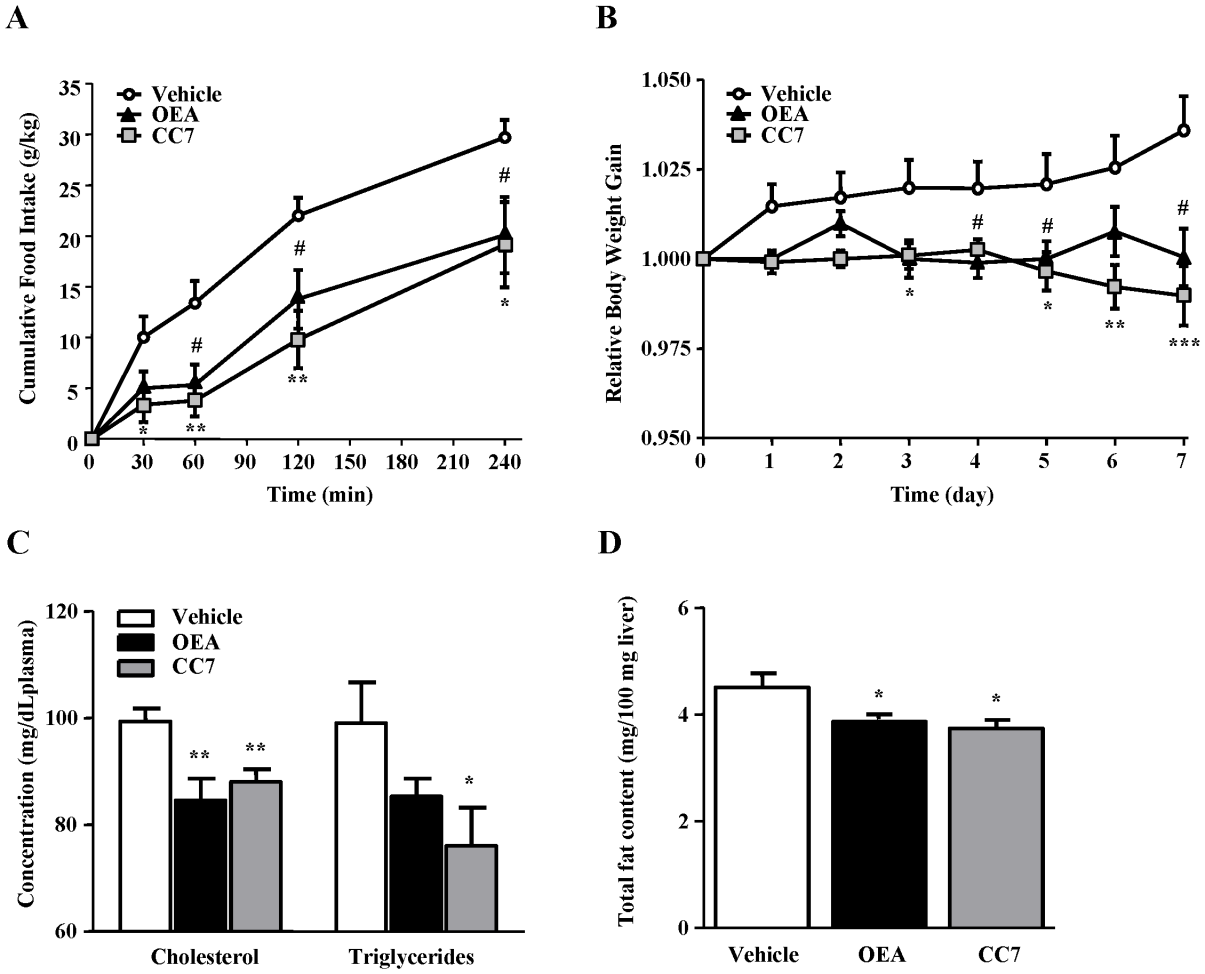
Effects of acute and subchronic treatment with OEA and CC7 (5 mg/kg, i.p.) on lipid metabolism and feeding behavior in Wistar rats. (**A**) Effects on food intake in rats deprived of food for 24 h after acute treatment with OEA and CC7 (N = 7). The data are represented as the mean ± SEM and were analyzed by two-way ANOVA and *post hoc* Bonferroni’s tests. #P<0.05, *P<0.05 and **P<0.01 compared to vehicle-treated rats. (**B**) Body weight gain after a 7-day treatment with OEA and CC7 in free-feeding rats (N = 8). The data are represented as the mean ± SEM and were analyzed by two-way ANOVA and *post hoc* Bonferroni’s tests. *P<0.05, **P<0.01, ***P<0.001 compared to vehicle-treated rats. (**C**) Cholesterol and triglyceride content in the plasma after a 7-day treatment with OEA and CC7 in free-feeding rats (N = 8). The bars represent the mean ± SEM. Data were analyzed by Student’s t test. **P<0.01 compared to vehicle-treated rats. (**D**) Total fat content in the liver after a 7-day treatment with OEA and CC7 in free-feeding rats (N = 8). The bars represent the mean ± SEM. Data were analyzed by Student’s t test. *P<0.05 compared to vehicle-treated rats.

To demonstrate the *in vivo* effects of OEA (5 mg/kg) and CC7 (5 mg/kg) as anorectic drugs on feeding behavior, we injected previously established doses [17], [31] in rats deprived of food for 24 h. As shown **Figure 6A**, acute treatment with both compounds induced a similar decrease in food intake compared to the vehicle group. Two-way ANOVA analysis of the acute effects on feeding behavior revealed that the different treatments produced a significant effect on food intake (F _2, 90_ = 16.85; P<0.0001). As expected, time was reflected in the increase of the cumulative food intake (F _4, 90_ = 47.20; P<0.0001). *Post-hoc* analysis indicated that both OEA and CC7 produced an appetite-suppressing effect, resulting in a significant decrease of food intake at different times (OEA: P<0.05 at 60, 120 and 240 min; CC7: P<0.05 at 30 and 240 min, P<0.01 at 60 and 120 min).

### Effects of Subchronic Treatment with OEA and CC7 in Wistar Rats

To further explore their anorectic properties, we tested both drugs in adult Wistar rats by administering subchronic treatment for 7 days. This extended treatment with either OEA or CC7 reduced body weight gain and improved the lipid profiles in the plasma and liver. The relative body weight gain was only affected by treatment (F _2, 168_ = 30.42; P<0.0001) because this variable was inhibited in OEA- and CC7-treated rats compared to control rats (**Fig. 6B**). *Post hoc* analysis showed significant decreases after 3–4 days of treatment with OEA (P<0.05, 3^rd^ and 5^th^ day; P<0.01, 6^th^ day; and P<0.001, 7^th^ day) and CC7 (P<0.05, 3^rd^ and 5^th^ day; P<0.01, 6^th^ day; and P<0.001, 7^th^ day) compared to vehicle-treated rats.

Additionally, we measured the circulating lipid parameters (**Fig. 6C**) and fat content in the liver (**Fig. 6D**). Both OEA and CC7 produced a significant decrease in the total cholesterol content in the plasma (P<0.01) compared to the vehicle group. Plasma triglyceride levels were also decreased in these animals, although only CC7 treatment resulted in a significant reduction (P<0.05) compared to vehicle-treated rats. Regarding the liver, OEA and CC7 significantly reduced the amount of fat deposition (P<0.05). These data suggest that both compounds act as hypolipidemic agents similar to fibrates.

### Effects of OEA and CC7 on the Writhing Test in Mice

We have been demonstrated that OEA has antinociceptive effects in a dose-dependent manner in CD1 mice [14]. We have also shown that these effects were similar in PPARα-deficient mice, suggesting that the nuclear receptors might not play an important role in these properties of OEA. For this reason, we repeated the same experiment with CC7.

**Figure 7:**
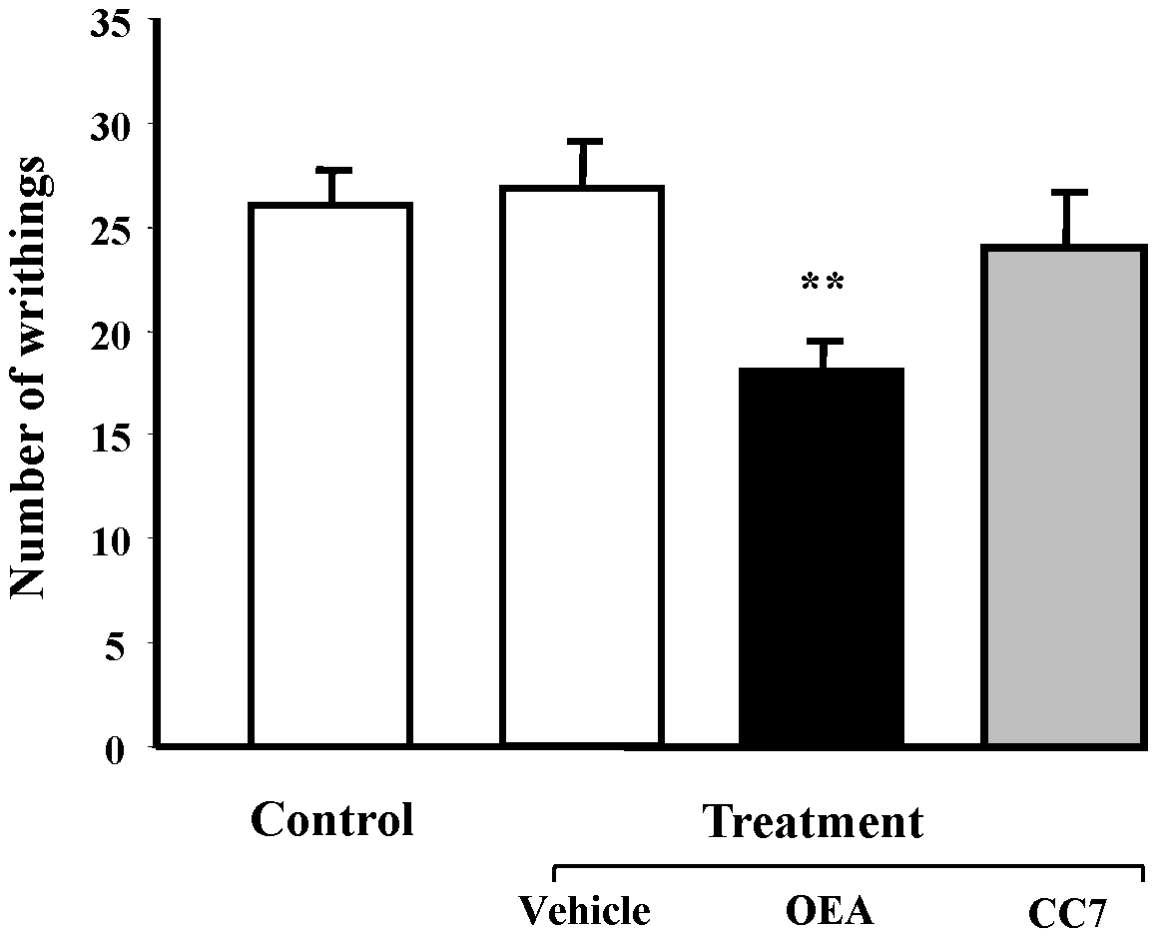
Effects of OEA and CC7 (5 mg/kg, i.p.) on abdominal constrictions (writhing) caused by i.p. injection of 0.6% acetic acid in CD1 mice. Drugs were administered 15± SEM. Data were analyzed by Student’s t test. **P<0.01 compared to vehicle- and acetic acid-treated mice.

Systemic injection of acetic acid (0.6%) evoked a stereotypical writhing response in CD1 mice. No differences were observed between the control mice and vehicle-treated animals. However, treatment with OEA decreasing the writhing response induced by acetic acid. Treatment with 5 mg/kg of OEA significantly inhibited the nociceptive response compared to the vehicle group (P<0.01) (**Fig. 7**). However, CC7 had no effect modifying the pain behavior evoked, which could indicate a lack of activity on other potential targets where OEA is able to act. See Supporting Information (Table S1 and Table S2).

## Discussion

In the present study, we have shown that N-octadecyl-N′-propylsulfamide, referred to as CC7, interacts with the nuclear receptor PPARα in the same manner as other PPARα agonists, such as OEA and GW7647. This common interaction with the ligand binding domain of PPARα (LBD- PPARα) was observed by docking studies and demonstrated by molecular biology and *in vitro* studies. CC7 and OEA exhibited a similar pattern to induce interactions between functional RXRα-PPARα heterodimers and target genes, resulting in enhanced binding to co-activators. Further, we have observed a PPARα-dependent induction of CPT1a mRNA as target gene in the presence of CC7 and OEA in human hepatic cells. We also conducted *in vivo* assays in rodents, and differential actions were observed for OEA compared to CC7. Both alkyl-based compounds displayed anorectic effects in rats as appetite-suppressing agents, weight gain inhibitors and hypolipidemic agents. However, whereas OEA was observed to be antinociceptive in mice, CC7 had no effects in visceral pain.

Molecular docking of the interaction of these compounds with the PPARα-LBD supported the stabilization of the agonistic conformation of the ligand-PPARα complex. The binding modes selected for GW7647, OEA and CC7 produced active conformations with a similar structural profile to GW409544 (the co-crystallized model). Different amino acids contributed to the stabilization of the active conformation of PPARα-LBD (Ser-280, His-440, Tyr-314 and Tyr-464) by directly stabilizing helix 12 via hydrogen bonds. Because these active conformations could facilitate the interaction of PPARα with co-activators, we performed studies with co-activators and target genes.

The transcriptional control of genes involved in lipid metabolism appears to be extremely complex, and the net effect of PPARα may depend upon heterodimerization (heterodimers with RXRα exhibit greater potential for trans-activation than either PPAR or RXR alone) and either binding or displacing nuclear factors that may have positive (co-activators) or negative (co-repressors) transcriptional influence [32]. To date, almost all of the PPREs identified in the rat and human genome that are inducible have a DR1 motif that binds PPARα-RXRα heterodimers with greater affinity.

It is well established that PPARα is a major transcriptional regulator of fatty acid metabolism in the liver [33]. Several genes involved in fatty acid oxidation and other metabolic processes have been identified as a direct target of PPARα through the presence of a functional PPRE (i.e., ACOX and CPT1) [34].

In this study, we initially showed by super-shift experiments that the presence of OEA induced effective PPARα-RXRα heterodimers that interacted with the PPRE of the ACOX gene. We also performed *in vitro* studies with the PPRE (DR1) of human CPT1 gene. Our results showed that the interaction of the co-activator SRC1 with PPARα through a functionally active PPRE in the CPT1 gene was stronger in the presence of either OEA or CC7 compared to CC12. Previously we reported a similar effect using another co-activator, TIF2 [17]. Complementary to the GST pull-down studies with CPT1, we performed additional *in vitro* studies in transfected cells with CPT1-PPRE (DR1)-luciferase and human PPARα and RXRα. Again, PPARα agonists such as OEA, CC7 and GW7647 induced an increase in luciferase activity by enhancing the interaction between CPT1-PPRE and PPARα-RXRα in presence of SRC1. To investigate how RXRα-PPARα modulated the acetylation status of histones and other factors associated with PPREs of target genes in the presence of these drugs, we transiently transfected MCF-7 cells with expression vectors for PPARα and RXRα in either the presence or absence of the HDAC inhibitor TSA. Our results showed for the first time that PPARα inhibition by co-repressors was completely abolished by TSA in the presence of OEA and other agonists, including CC7 and GW7647. This finding rules out the enhanced recruitment of HDACs as the mechanism of PPAR inhibition in presence of NCoR. As confirmation, we performed translational studies demonstrating that CC7 and OEA induced the mRNA expression of CPT1a in hepatic cells through PPARα regulation, since specific siRNA for PPARα prevented such inductive effects on CPT1a mRNA.

We have previously reported that OEA and some sulfamoyl-derived analogs (including CC7) are able to activate PPARα in a dose-dependent manner with the same biological activity as appetite suppressants [17], [31]. Here, we demonstrated this satiety effect on feeding behavior after acute injections of OEA and CC7 in fasted Wistar rats. We reported that daily injections of CC7 prevented body weight gain in obese Zucker and Wistar rats [17], whereas OEA reduced the body weight gain in both obese and lean Zucker rats [31] using different doses in independent paradigms. We also performed a 7-day treatment with 5 mg/kg of both alkyl-based compounds and observed identical profiles. As expected, our data showed a pronounced reduction in body weight gain in Wistar rats starting on the 3^rd^ day, which could be due to the satiety effects described in acute treatments for both drugs and their actions on the catabolism of lipid metabolism via PPARα. This was confirmed after evaluating the plasma lipid parameters and hepatic fat content because the cholesterol levels and triglycerides were decreased in the plasma, and fat content was also reduced after treatment with either OEA or CC7. Additionally, we showed that these *in vivo* effects of CC7 on feeding behavior were not related to a cannabinoid mechanism since CC7 is not ligand for CB_1_ and also not interfere with the cannabinoid degradation (**Table S1**). Therefore, both drugs act as hypolipidemic agents in a similar manner to fibrates, although the side effects on the kidneys and muscle should be evaluated. Nevertheless, theoretical pharmacokinetics studies were also performed and predicted that both compounds could act as drugs without bioavailability impediments or toxicity issues (**Table S2**).

Nevertheless, OEA has been demonstrated to act through multiple mechanisms and targets independent of PPARα, which might explain the variety of actions displayed by this NAE [8]. For example, our group reported that OEA reduces visceral and inflammatory responses through a PPARα-independent mechanism in mice [14]. Considering the similar effects in feeding behavior and lipid metabolism of OEA and CC7, we evaluated their actions on the nociceptive response. The observed lack of activity of CC7 on visceral analgesia, a pharmacological action of OEA not mediated by PPARα, clearly supports the hypothesis that OEA (but not CC7) can signal through targets other than PPARα and that these pathways can be functionally dissociated from the metabolic PPARα-dependent pathways. In any case, other potential targets for CC7 should be assessed including the capsaicin receptor (TRPV1) and GPR119 to confirm this specificity for PPARα, since OEA has been reported that activates these receptors [15]–[16].

In conclusion, OEA and CC7 are able to act similarly on the ligand binding domain of PPARα, as suggested by the computational docking results and transcriptional studies. Moreover, both compounds display anorexigenic and lipid-reducing properties, which are mediated by PPARα. However, CC7 has no analgesic effects, unlike OEA, which is able to produce anti-nociceptive effects through an unknown mechanism. These observations suggest that CC7 might represent a novel pharmacological tool for the treatment of obesity because it lacks some of properties of other N-acylethanolamines in pain and inflammation.

## Supporting Information

Table S1 **Structures and binding data of compounds.**
(DOCX)

Table S2 **Pharmacokinetic and theoretical parameters related to the bioavailability of CC7 and OEA.**
(DOCX)

Material and Methods S1 **Material and methods for supplementary Tables S1 and S2. (A)** (CB_1_) Radioligand Binding Assays. (B) FAAH Assays. (C) *In silico* pre-ADMET study.
(DOCX)
