# Supplementary material for: Computational and Biological Evaluation of N-octadecyl-N′-propylsulfamide, a Selective PPARα Agonist Structurally Related to N-acylethanolamines
Source: PLoS One. 2014 Mar 20;9(3):e92195. doi: 10.1371/journal.pone.0092195 (PMC3961330; doi:10.1371/journal.pone.0092195)
Supplement: Table S1 — Structures and binding data of compounds. (DOCX) [file pone.0092195.s001.docx]

**Table S1.** Structures and binding data of compounds

| **Compound** | **R1 group** | **Sulfamide** | **R2 group** | ***K*i (nM)^a^** | **FAAH inhibition at 10µM**  **(% control) ^b^** |
| --- | --- | --- | --- | --- | --- |
| ***N*-octadecyl sulfamide** | octadecane (C_18_H_38_) | (R1) H_2_NSO_2_NH_2_ (R2) | H | >10,000 | -7.49 |
| ***N*-adamantyl sulfamide** | adamantane (C_10_H_16_) |  | H | >10,000 | -0.95 |
| **CC7**  ***N*-octadecyl-*N´*-propylsulfamide** | octadecane (C_18_H_38_) |  | Propane  (C_3_H_8_) | 9,102± 140 | -8.28 |
| **CC12**  ***N*-adamantyl-*N´*-propylsulfamide** | adamantane (C_10_H_16_) |  | Propane  (C_3_H_8_) | 6,670± 1,320 | 9.61 |
| **Anandamide**  ***N-*arachidonoylethanolamine** |  |  |  | 132± 23 |  |

^a^ Affinity of compounds for the CB_1_ receptor was evaluated using rat cerebellum membrane and [^3^H]SR141716A.

^b^ FAAH activity was evaluated using rat cerebral membrane and 10 µM of [^3^H]AEA.
