# Supplementary material for: Computational and Biological Evaluation of N-octadecyl-N′-propylsulfamide, a Selective PPARα Agonist Structurally Related to N-acylethanolamines
Source: PLoS One. 2014 Mar 20;9(3):e92195. doi: 10.1371/journal.pone.0092195 (PMC3961330; doi:10.1371/journal.pone.0092195)
Supplement: Table S2 — Pharmacokinetic and theoretical parameters related to the bioavailability of CC7 and OEA. (DOCX) [file pone.0092195.s002.docx]

**Table S2.** Pharmacokinetic and theoretical parameters related to the bioavailability of CC7 and OEA.

| **Parameters/Properties** | **Lipinski’s rule** | **CC7** | **OEA** |
| --- | --- | --- | --- |
| **cLogP** | **≤ 5** | 7.41 | 6.56 |
| **MW** | **≤ 500** | 376 | 325 |
| **# Hydrogen bond acceptors** | **≤ 10** | 4 | 3 |
| **# Hydrogen bond donors** | **≤ 5** | 2 | 2 |
| **Lipinski’s violations** | **≤ 1** | 1 | 1 |
| **PSA** (absorption-transport ≤ 83) | - | 58.2 | 49.3 |
| **Log BB** (blood-brain barrier transport ≥ 0.5) | - | 0.73 | 0.68 |

cLogP: logarithm of partition coefficient between n-octanol and water; MW: molecular weight; PSA: polar surface area; Log BB: logarithm of blood brain portioning.
