# Supplementary material for: Computational and Biological Evaluation of N-octadecyl-N′-propylsulfamide, a Selective PPARα Agonist Structurally Related to N-acylethanolamines
Source: PLoS One. 2014 Mar 20;9(3):e92195. doi: 10.1371/journal.pone.0092195 (PMC3961330; doi:10.1371/journal.pone.0092195)
Supplement: Material and Methods S1 — Material and methods for supplementary Tables S1 and S2. (A) (CB1) Radioligand Binding Assays. (B) FAAH Assays. (C) In silico pre-ADMET study. (DOCX) [file pone.0092195.s003.docx]

**MATERIAL AND METHODS S1**

**A) (CB_1_) Radioligand Binding Assays** (for **Table S1**)

A P2 membrane fraction from rat cerebellar homogenates was prepared. The CB_1_ (cannabinoid receptor type 1) binding assays in rat cerebellar membranes were performed using [^3^H]SR141716A (NEN-Dupont, Boston, MA, 40-60 Ci/mmol) as ligand. Assays were performed for 60-90 min at 30°C in a final volume of 0.5 mL. For competition analysis, drugs were dissolved in 100% DMSO to a final concentration of 10 nM. Further dilutions were made in assay buffer to reach concentrations spanning between 10^-5^ to 10^-12^ M (final concentration in tubes). *K*_i_ for the different drugs assayed were calculated from the equation of Cheng and Prusoff, using fixed *K*d values for ^3^[H]SR141716A obtained from independent experimental assays [1].

**B) FAAH Assays** (for **Table S1**)

Brain tissues were homogenized in 50 mM Tris buffer, pH 8, containing 0.32 M sucrose. Homogenates were centrifuged first at 1,000 × g (5 min), the pellet discarded and the supernantant centrifuged at 45,000 × g (30 min). The pellets obtained were solubilized at 0-4°C in Tris buffer. Protein content in the membrane fraction was measured with the Bradford method. All membrane fractions were stored at -70ºC until used. The enzymatic assay was run under conditions that were linear with time and protein concentration. We assayed membrane-bound amidehydrolase (FAAH) activity using N-arachidonoyl-[1-^3^H]-ethanolamine ([^3^H]AEA) as a substrate, and measuring metabolized AEA as [^3^H]ethanolamine in the aqueous phase after chloroform extraction, as described [2-3]. Briefly: Standard FAAH assays were carried out for 10 min at 37ºC in 1 mL of TRIS buffer (50mM, pH 7.5) containing membrane fraction (100 mg of protein) and a saturating 10 µM concentration of [^3^H]AEA (10,000 dpm/mL) with Ultima Gold scintillation liquid (Perkin Elmer, Waltham, MA, USA). Identical incubations were performed in the absence of tissue: these ‘control’ samples contained around 80-95 dpm/sample that were subtracted from values obtained with tissue samples. Determinations of disintegrations were measured using a Beckman LS6500 scintillation counter. Results were expressed as % of FAAH activity vs. membrane non-treated.

**C) *In silico* Pre-ADMET Study** (for **Table S2**)

The Molinspiration Cheminformatics website (http://www.molinspiration.com/; accessed 10/07/2013) was used to perform the theoretical oral bioavailability study analyzing the molecular properties of the Lipinski rule (cLogP: logarithm of the partition coefficient between n-octanol and water; MW: molecular weight; HBA: number of hydrogen bond acceptors; and HBD: number of hydrogen bond donors). To predict the blood-brain barrier permeability, we employed the theoretical logarithm of blood brain portioning (logBB), which uses the polar surface area (PSA) and the clogP as descriptors. Finally, a theoretical toxicity study was performed using the Osiris Property Explorer (http://www.organic-chemistry.org/prog/peo/; accessed 09/08/2013).

**Bibliography**

1. Cheng Y, Prusoff WH (1973) Relationship between the inhibition constant (K1) and the concentration of inhibitor which causes 50 per cent inhibition (I50) of an enzymatic reaction. Biochem Pharmacol 22: 3099-3108.

2. Desarnaud F, Cadas H, Piomelli D (1995) Anandamide amidohydrolase activity in rat brain microsomes. Identification and partial characterization. J Biol Chem 270: 6030-6035.

3. Hansson AC, Bermudez-Silva FJ, Malinen H, Hyytia P, Sanchez-Vera I, et al. (2007) Genetic impairment of frontocortical endocannabinoid degradation and high alcohol preference. Neuropsychopharmacology 32: 117-126.
